# Planting the Seeds of a New Paradigm

**DOI:** 10.1371/journal.pbio.0020133

**Published:** 2004-05-11

**Authors:** Marjori A Matzke, Antonius J. M Matzke

## Abstract

RNA-mediated gene silencing has emerged in recent years as an important mechanism for regulating gene expression. Some of the key discoveries have been made in plants

Although the word ‘revolution’ should not be used lightly in science, there is no other way to describe the recent explosion in our awareness and understanding of RNA-mediated gene silencing pathways. The central player in RNA-mediated gene silencing is a double-stranded RNA (dsRNA) that is chopped into tiny RNAs by the enzyme Dicer. The tiny RNAs associate with various silencing effector complexes and attach to homologous target sequences (RNA or DNA) by basepairing. Depending on the protein composition of the effector complex and the nature of the target sequence, the outcome can be either mRNA degradation, translational repression, or genome modification, all of which silence gene expression (Figure 1). Present in plants, animals, and many fungi, RNA-mediated gene silencing pathways have essential roles in development, chromosome structure, and virus resistance. Although the mechanistic details are still under investigation, RNA-mediated silencing has already provided a powerful tool for studying gene function and spawned a fledgling industry that aims to develop novel RNA-based therapeutics to treat human diseases (Robinson 2004).

**Figure 1:**
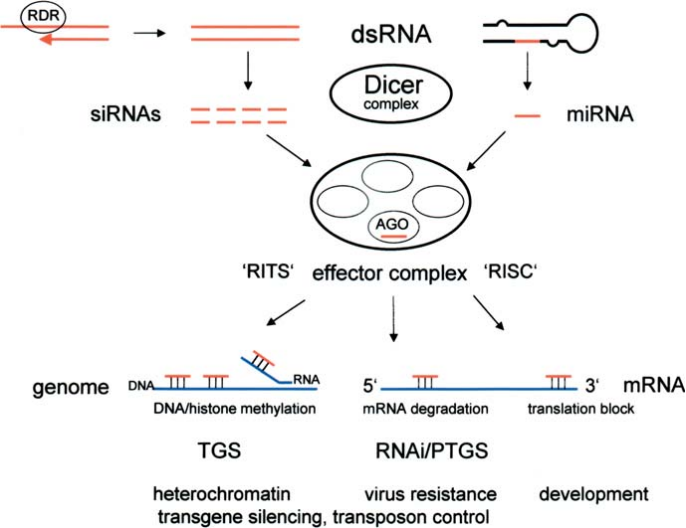
RNA-Mediated Silencing Short RNAs derived from Dicer cleavage of dsRNA are incorporated into multiprotein effector complexes, such as RISC and RITS (RNA-induced initiation of TGS) (Verdel et al. 2004) to target mRNA degradation (RNAi/PTGS), translation inhibition, or TGS and genome modifications. ARGONAUTE (AGO) proteins (the name comes from a plant mutant [Bohmert et al. 1998]) bind short RNAs and ‘shepherd’ them to appropriate effector complexes (Carmell et al. 2002). siRNAs originate from perfect RNA duplexes, which can be produced by RDR activity on ssRNA templates; miRNAs originate from imperfect RNA hairpins that are encoded in intergenic regions of plant and animal genomes. Functions are shown at the bottom. In addition to roles in transgene silencing, both TGS and RNAi/PTGS control genome parasites called transposons (Flavell 1994; Plasterk 2002). Genome modifications (DNA and histone methylation) can potentially be targeted by short RNAs that basepair to DNA or to nascent RNA synthesized from the target gene (Grewal and Moazed 2003). Target nucleic acids are shown in blue, short RNAs in red, proteins and enzyme complexes as ovals.

Many biologists first learned of RNA-mediated gene silencing in 1998 following the discovery, in the nematode worm Caenorhabditis elegans (Fire et al. 1998), of a process called RNA interference (RNAi), in which dsRNA triggers sequence-specific mRNA degradation. The roots of RNA-mediated silencing, however, can be traced back 15 years, when a handful of botanical labs stumbled across strange cases of gene silencing in transgenic plants. To highlight the many seminal contributions of plant scientists to the field, we offer here a personal perspective on the origins and history of RNA-mediated gene silencing in plants.

## Early Silencing Phenomena

Starting in the late 1980s, biologists working with transgenic plants found themselves confronted with a ‘bewildering array’ of unanticipated gene silencing phenomena (Martienssen and Richards 1995). Most intriguing were cases in which silencing seemed to be triggered by DNA or RNA sequence interactions, which could occur between two separate transgenes that shared sequence homology or between a transgene and homologous plant gene. Several early examples supplied the prototypes for two types of RNA-mediated gene silencing that are recognized today. In one type, silencing results from a block in mRNA synthesis (transcriptional gene silencing [TGS]); in the second type, silencing results from mRNA degradation (posttranscriptional gene silencing [PTGS]) (Figure 1).

**Figure 2:**
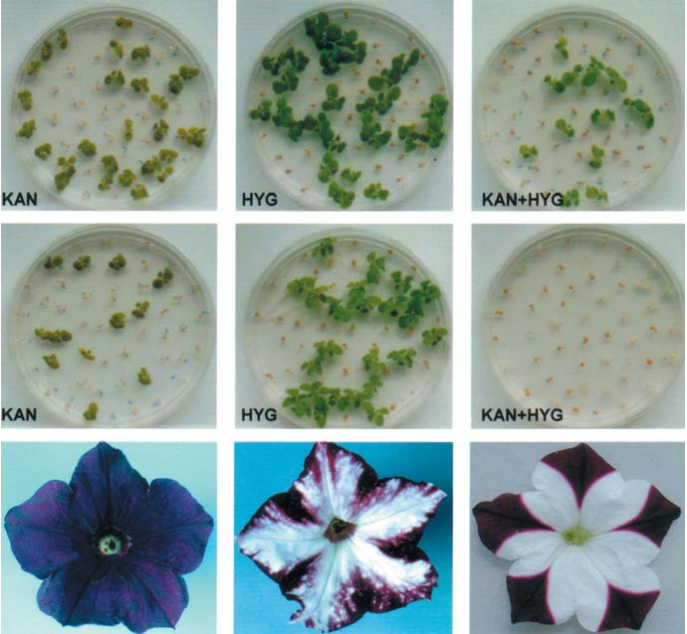
Early Examples of Gene Silencing in Transgenic Plants TGS: Normally when two plants harboring separate transgenes encoding resistance to kanamycin (kan) or hygromycin (hyg), respectively, are crossed, 50% of the progeny are resistant to the individual antibiotics and 25% are resistant to a combination of both (top). In cases of silencing, expression of the KAN marker is extinguished in the presence of the HYG marker, as indicated by only 25% kan resistance and no double resistance (middle). PTGS: Transformation of wild-type petunia (bottom left) with a transgene encoding a pigment protein can lead to loss of pigment (white areas) owing to cosuppression of the transgene and homologous endogenous plant gene. (Photos on the left and in the middle were provided by Jan Kooter and on the right were provided by Natalie Doetsch and Rich Jorgensen.)

TGS was revealed when two different transgene complexes were introduced in sequential steps into the tobacco genome. Each complex encoded different proteins, but contained identical gene regulatory regions (promoters). Unexpectedly, the first transgene complex, which was stably active on its own, often became silenced in the presence of the second (Figure 2). The promoters of the silenced transgenes acquired DNA methylation, a genome modification frequently associated with silencing. Silencing and methylation were reversed when the transgene complexes segregated from each other in progeny, suggesting that interactions between the common promoter regions triggered silencing and methylation (Matzke et al. 1989; Park et al. 1996).

PTGS was discovered in two ways. One involved experiments to evaluate antisense suppression, a promising approach at the time for selectively silencing plant gene expression. In theory, antisense RNA encoded by a transgene should basepair to the complementary mRNA of a plant gene, preventing its translation into protein. Although the control ‘sense’ transgene RNAs are unable to basepair to mRNA and hence should not induce silencing, they often inexplicably did (Smith et al. 1990). In another type of experiment, efforts to enhance floral coloration in petunia by overexpressing a transgene encoding a protein involved in pigment synthesis led paradoxically to partial or complete loss of color (Figure 2). This resulted from coordinate silencing (‘cosuppression’) of both the transgene and the homologous plant gene (Napoli et al. 1990; Van der Krol et al. 1990), later shown to occur at the posttranscriptional level (De Carvalho et al. 1992; Van Blokland et al. 1994) A related phenomenon, called quelling, was observed in the filamentous fungus Neurospora crassa (Romano and Macino 1992). Similarly to TGS, PTGS was often associated with DNA methylation of transgene sequences (Ingelbrecht et al. 1994).

Two influential papers appeared in the early 1990s. One reported the discovery of RNA-directed DNA methylation in transgenic tobacco plants (Wassenegger et al. 1994). This was the earliest demonstration of RNA-induced modification of DNA, a process that we return to below. A second study showed that plant RNA viruses could be both initiators and targets of PTGS. Plants expressing a transgene encoding a truncated viral coat protein became resistant to the corresponding virus, a state achieved by mutual degradation of viral RNA and transgene mRNA (Lindbo et al. 1993). In addition to forging a link between RNA virus resistance and PTGS, this study included a remarkably prescient model for PTGS that featured an RNA-dependent RNA polymerase (RDR), small RNAs, and dsRNA, all of which were later found to be important for the RNAi. PTGS was subsequently shown in 1997 to protect plants naturally from virus infection (Covey et al. 1997; Ratcliff et al. 1997). Transgene PTGS thus tapped into a preexisting natural mechanism for combating viruses.

To recap: by 1998—the year in which RNAi was reported—plant scientists had documented sequence-specific RNA degradation (PTGS), sequence-specific DNA methylation that triggered TGS, and RNA-directed DNA methylation. They had also proposed models for PTGS involving dsRNA (Lindbo et al. 1993; Metzlaff et al. 1997), small RNAs, and RDR (Lindbo et al. 1993).

## RNAi

RNAi was discovered in experiments designed to compare the silencing activity of single-stranded RNAs (ssRNAs) (antisense or sense) with their dsRNA hybrid. While only marginal silencing of a target gene was achieved after injecting worms with the individual strands, injection of a sense–antisense mixture resulted in potent and specific silencing (Fire et al. 1998). This unequivocally fingered dsRNA as the trigger of silencing. Shortly thereafter, dsRNA was shown to provoke gene silencing in other organisms, including plants (Waterhouse et al. 1998). Indeed, the relatedness of RNAi, PTGS, and quelling was confirmed when genetic analyses in worms, plants, and Neurospora identified common components in the respective silencing pathways (Denli and Hannon 2003). This included the aforementioned RDR, which can synthesize dsRNA from ssRNA templates (see Figure 1). PTGS is now accepted as the plant equivalent of RNAi.

The discovery of RNAi established a requirement for dsRNA in silencing, but details of the mechanism remained unclear. In 1999, plant scientists studying PTGS provided a crucial clue when they detected small (approximately 25 nucleotide-long) RNAs corresponding to silenced target genes in transgenic plants (Hamilton and Baulcombe 1999). They proposed that the small RNAs provided the all-important specificity determinant for silencing. Consistent with this, a rapid succession of studies in Drosophila systems demonstrated that 21–23 nucleotide ‘short interfering'RNAs (siRNAs), derived from cutting longer dsRNA, can guide mRNA cleavage (Zamore et al. 2000; Elbashir et al. 2001); identified RISC (RNA-induced silencing complex), a nuclease that associates with small RNAs and executes target mRNA cleavage (Hammond et al. 2000); and identified Dicer, the enzyme that chops dsRNA into short RNAs (Bernstein et al. 2001) (see Figure 1).

RNAi/PTGS was detected originally in experiments involving transgenes, injected RNAs, or viruses. Did the RNAi machinery also generate small RNAs for host gene regulation? Strikingly, the newly discovered siRNAs were the same size as several ‘small temporal’ RNAs, first identified in 1993 as important regulators of developmental timing in worms (Lee et al. 1993; Reinhart et al. 2000). Everything came together in 2001 when heroic cloning efforts unearthed dozens of natural small RNAs 21–25 nucleotides in length, first from worms and flies and later from plants and mammals (Lai 2003; Bartel 2004). Similar to siRNAs, the natural small RNAs, dubbed microRNAs (miRNAs), arise from Dicer processing of dsRNA precursors and are incorporated into RISC (Denli and Hannon 2003). In many cases, miRNAs effect silencing by basepairing to the 3′ ends of target mRNAs and repressing translation (see Figure 1). miRNAs are now recognized as key regulators of plant and animal development. Identifying their target genes and full range of action are areas of intense research (Lai 2003; Bartel 2004).

Up until 2002, RNAi/PTGS and miRNAs were the most avidly studied aspects of RNA-mediated gene silencing. The next major advance, however, abruptly turned attention back to RNA-guided modifications of the genome. By 2001, plant scientists working on RNA-directed DNA methylation and TGS had demonstrated a requirement for dsRNAs that are processed to short RNAs, reinforcing a mechanistic link to PTGS (Mette et al. 2000; Sijen et al. 2001). This established the principle of RNA-guided genome modifications, but the generality of this process was uncertain because not all organisms methylate their DNA. Widespread acceptance came with the discovery in 2002 of RNAimediated heterchromatin assembly in fission yeast (Hall et al. 2002; Volpe et al. 2002). This silencing pathway uses short RNAs produced by Dicer and other RNAi components to direct methylation of DNA-associated proteins (histones), thus generating condensed, transcriptionally silent chromosome regions (heterochromatin) (see Figure 1). Targets of this pathway include centromeres, which are essential for normal chromosome segregation. The RNAi-dependent heterochromatin pathway has been found in plants (Zilberman et al. 2003) and Drosophila (Pal-Bhadra et al. 2004) and likely represents a general means for creating condensed, silent chromosome domains.

## More Lessons from Plants

Plant scientists can chalk up other ‘firsts’ in RNA-mediated gene silencing. Systemic silencing, in which a silencing signal (short RNA or dsRNA) moves from cell to cell and through the vascular system to induce silencing at distant sites, was initially detected in plants in 1997 (Palauqui et al. 1997; Voinnet and Baulcombe 1997) and later in worms (Fire et al. 1998), although not yet in Drosophila or mammals. Viral proteins that suppress silencing by disarming the PTGS-based antiviral defense mechanism were discovered by plant virologists in 1998 (Anandalakshmi et al. 1998; Béclin et al. 1998; Brigneti et al. 1998; Kasschau and Carrington 1998). One of these, the p19 protein of tombusviruses, acts as a size-selective caliper to sequester short RNAs from the silencing machinery (Vargason et al. 2003). A recent study suggests that animal viruses encode suppressors of RNA-mediated silencing (Li et al. 2004).

Although RNA-mediated gene silencing pathways are evolutionarily conserved, there are various elaborations in different organisms. For example, the plant Arabidopsis has four Dicer-like (DCL) proteins, in contrast to mammals and worms, whose genomes encode only one Dicer protein (Schauer et al. 2002). The RDR family has also expanded in Arabidopsis to include at least three active members. An important goal has been to determine the functions of individual family members. Previous studies in Arabidopsis have shown that DCL1 is needed for processing miRNA precursors important for plant development (Park et al. 2002; Reinhart et al. 2002), but not for siRNAs active in RNAi (Finnegan et al. 2003). The paper by Xie et al. (2004) in this issue of *PLoS Biology* delineates distinct functions for DCL2, DCL3, and RDR2. Nuclear-localized DCL3 acts with RDR2 to generate short RNAs that elicit DNA and histone modifications; DCL2 produces short RNAs active in antiviral defense in the cytoplasm of cells. This study illustrates nicely how RNA silencing components have diversified in plants to carry out specialized functions.

By identifying small RNAs as agents of gene silencing that act at multiple levels throughout the cell, molecular biologists have created a new paradigm for eukaryotic gene regulation. Plant scientists have figured prominently in RNA-mediated silencing research. Instrumental to their success was the early ability to produce large numbers of transgenic plants, which displayed a rich variety of gene silencing phenomena that were amenable to analysis. The agricultural biotechnology industry provided incentives to find ways to stabilize transgene expression and use transgenic approaches to modulate plant gene expression and to genetically engineer virus resistance. As exemplified by the petunia cosuppression experiments, nonessential plant pigments provide conspicuous visual markers that vividly reveal gene silencing. The history of gene silencing research shows once again that plants offer outstanding experimental systems for elucidating general biological principles.

## Abbreviations

AGO: ARGONAUTE
DCL: Dicer-like
dsRNA: double-stranded RNA
miRNA: microRNA
PTGS: posttranscriptional gene silencing
RDR: RNAdependent RNA polymerase
RISC: RNA-induced silencing complex
RITS: RNA-induced initiation of transcriptional gene silencing
RNAi: RNA interference
siRNA: short interfering RNA
ssRNA: single-stranded RNA
TGS: transcriptional gene silencing

## References

[pbio-0020133-Anandalakshmi1] Anandalakshmi R, Pruss G, Ge X, Marathe R, Mallory A (1998). A viral suppressor of gene silencing in plants. Proc Natl Acad Sci U S A.

[pbio-0020133-Bartel1] Bartel DP (2004). MicroRNAs: Genomics, biogenesis, mechanism and function. Cell.

[pbio-0020133-Beclin1] Béclin C, Berthomé R, Palauqui J, Tepfer M, Vaucheret H (1998). Infection of tobacco or Arabidopsis plants by CMV counteracts systemic post-transcriptional silencing of nonviral (trans)genes. Virology.

[pbio-0020133-Bernstein1] Bernstein E, Caudy A, Hammond S, Hannon G (2001). Role for a bidentate ribonuclease in the initiation step of RNA interference. Nature.

[pbio-0020133-Bohmert1] Bohmert K, Camus I, Bellini C, Bouchez D, Caboche M (1998). AGO1 defines a novel locus of Arabidopsis controlling leaf development. EMBO J.

[pbio-0020133-Brigneti1] Brigneti G, Voinnet O, Li W-X, Ji L-H, Ding S-W (1998). Viral pathogenicity determinants of transgene silencing in Nicotiana benthamiana. EMBO J.

[pbio-0020133-Carmell1] Carmell M, Xuan Z, Zhang M, Hannon G (2002). The Argonaute family: Tentacles that reach into RNAi, developmental control, stem cell maintenance, and tumorigenesis. Genes Dev.

[pbio-0020133-Covey1] Covey S, Al-Kaff N, Lángara A, Turner D (1997). Plants combat infection by gene silencing. Nature.

[pbio-0020133-DeCarvalho1] De Carvalho F, Gheyson G, Kushnir S, van Montagu M, Inzé D (1992). Suppression of β-1,3-glucanase transgene expression in homozygous plants. EMBO J.

[pbio-0020133-Denli1] Denli A, Hannon G (2003). RNAi: An ever-growing puzzle. Trends Biochem Sci.

[pbio-0020133-Elbashir1] Elbashir S, Lendeckel W, Tuschl T (2001). RNA interference is mediated by 21- and 22-nucleotide RNAs. Genes Dev.

[pbio-0020133-Finnegan1] Finnegan EJ, Margis R, Waterhouse P (2003). Posttranscriptional gene silencing is not compromised in the Arabidopsis CARPEL FACTORY (DICER-LIKE1) mutant, a homolog of Dicer-1 from Drosophila. Curr Biol.

[pbio-0020133-Fire1] Fire A, Xu S, Montgomery M, Kostas S, Driver S (1998). Potent and specific genetic interference by double-stranded RNA in Caenorhabditis elegans. Nature.

[pbio-0020133-Flavell1] Flavell RB (1994). Inactivation of gene expression in plants as a consequence of specific sequence duplication. Proc Natl Acad Sci U S A.

[pbio-0020133-Grewal1] Grewal SIS, Moazed D (2003). Heterochromatin and epigenetic control of gene expression. Science.

[pbio-0020133-Hall1] Hall I, Shankaranarayana G, Noma K-I, Ayoub N, Cohen A (2002). Establishment and maintenance of a heterochromatic domain. Science.

[pbio-0020133-Hamilton1] Hamilton AJ, Baulcombe DC (1999). A species of small antisense RNA in posttranscriptional gene silencing in plants. Science.

[pbio-0020133-Hammond1] Hammond S, Bernstein E, Beach D, Hannon G (2000). An RNA-directed nuclease mediates post-transcriptional gene silencing in Drosophila cells. Nature.

[pbio-0020133-Ingelbrecht1] Ingelbrecht I, van Houdt H, Montagu M, Depicker A (1994). Posttranscriptional silencing of reporter transgenes in tobacco correlates with DNA methylation. Proc Natl Acad Sci U S A.

[pbio-0020133-Kasschau1] Kasschau K, Carrington J (1998). A counterdefensive strategy of plant viruses: Suppression of posttranscriptional gene silencing. Cell.

[pbio-0020133-Lai1] Lai EC (2003). microRNAs: Runts of the genome assert themselves. Curr Biol.

[pbio-0020133-Lee1] Lee R, Feinbaum R, Ambros V (1993). The C. elegans heterochronic gene *lin-4* encodes small RNAs with antisense complementarity to *lin-14*. Cell.

[pbio-0020133-Li1] Li W, Li H, Lu R, Li F, Dus M (2004). Interferon antagonist proteins of influenza and vaccinia viruses are suppressors of RNA silencing. Proc Natl Acad Sci U S A.

[pbio-0020133-Lindbo1] Lindbo J, Silva-Rosales L, Proebsting W, Dougherty W (1993). Induction of a highly specific antiviral state in transgenic plants: Implications for regulation of gene expression and virus resistance. Plant Cell.

[pbio-0020133-Martienssen1] Martienssen RA, Richards EJ (1995). DNA methylation in eukaryotes. Curr Opin Genet Dev.

[pbio-0020133-Matzke1] Matzke M, Primig M, Trnovsky J, Matzke A (1989). Reversible methylation and inactivation of marker genes in sequentially transformed plants. EMBO J.

[pbio-0020133-Mette1] Mette MF, Aufsatz W, van der Winden J, Matzke M, Matzke A (2000). Transcriptional gene silencing and promoter methylation triggered by double stranded RNA. EMBO J.

[pbio-0020133-Metzlaff1] Metzlaff M, O'Dell M, Cluster P, Flavell R (1997). RNA-mediated RNA degradation and chalcone synthase A silencing in petunia. Cell.

[pbio-0020133-Napoli1] Napoli C, Lemieux C, Jorgensen R (1990). Introduction of a chimeric chalcone synthase gene into petunia results in reversible cosuppression of homologous genes *in trans*. Plant Cell.

[pbio-0020133-Palauqui1] Palauqui J, Elmayan T, Pollien J, Vaucheret H (1997). Systemic acquired silencing: Transgene-specific post-transcriptional silencing is transmitted by grafting from silenced stocks to non-silenced scions. EMBO J.

[pbio-0020133-Pal-Bhadra1] Pal-Bhadra M, Leibovitch B, Gandhi S, Rao M, Bhadra U (2004). Heterochromatic silencing and HP1 localization in Drosophila are dependent on the RNAi machinery. Science.

[pbio-0020133-Park1] Park W, Li J, Song R, Messing J, Chen X (2002). CARPEL FACTORY, a Dicer homolog, and HEN1, a novel protein, act in microRNA metabolism in Arabidopsis thaliana. Curr Biol.

[pbio-0020133-Park2] Park Y-D, Papp I, Moscone E, Iglesias V, Vaucheret H (1996). Gene silencing mediated by promoter homology occurs at the level of transcription and results in meiotically heritable alterations in methylation and gene activity. Plant J.

[pbio-0020133-Plasterk1] Plasterk R (2002). RNA silencing: The genome's immune system. Science.

[pbio-0020133-Ratcliff1] Ratcliff F, Harrison B, Baulcombe D (1997). A similarity between viral defense and gene silencing in plants. Science.

[pbio-0020133-Reinhart1] Reinhart B, Slack F, Basson M, Pasquinelli A, Bettinger J (2000). The 21-nucleotide *let-7* RNA regulates developmental timing in Caenorhabditis elegans. Nature.

[pbio-0020133-Reinhart2] Reinhart B, Weinstein E, Rhoades M, Bartel B, Bartel D (2002). MicroRNAs in plants. Genes Dev.

[pbio-0020133-Robinson1] Robinson R (2004). RNAi therapeutics: How likely, how soon?. PLoS Biol.

[pbio-0020133-Romano1] Romano N, Macino G (1992). Quelling: Transient inactivation of gene expression in Neurospora crassa by transformation with homologous sequences. Mol Microbiol.

[pbio-0020133-Schauer1] Schauer S, Jacobsen S, Meinke D, Ray A (2002). DICER-LIKE1: Blind men and elephants in Arabidopsis development. Trends Plant Sci.

[pbio-0020133-Sijen1] Sijen T, Vijn I, Rebocho A, van Blokland R, Roelofs D (2001). Transcriptional and posttranscriptional gene silencing are mechanistically related. Curr Biol.

[pbio-0020133-Smith1] Smith C, Watson C, Bird C, Ray J, Schuch W (1990). Expression of a truncated tomato polygalacturonase gene inhibits expression of the endogenous gene in transgenic plants. Mol Gen Genet.

[pbio-0020133-VanBlokland1] Van Blokland R, van der Geest N, Mol J, Kooter J (1994). Transgene-mediated suppression of chalcone synthase expression in Petunia hybrida results from an increase in RNA turnover. Plant J.

[pbio-0020133-VanderKrol2] Van der Krol A, Mur L, Beld M, Mol JNM, Stuitje AR (1990). Flavonoid genes in petunia: Addition of a limited number of gene copies may lead to a suppression of gene expression. Plant Cell.

[pbio-0020133-Vargason1] Vargason J, Szittya G, Burgyán J, Tanaka Hall T (2003). Size selective recognition of siRNA by an RNA silencing suppressor. Cell.

[pbio-0020133-Verdel1] Verdel A, Jia S, Gerber S, Sugiyama T, Gygi S (2004). RNAi-mediated targeting of heterochromatin by the RITS complex. Science.

[pbio-0020133-Voinnet1] Voinnet O, Baulcombe D (1997). Systemic silencing in gene silencing. Nature.

[pbio-0020133-Volpe1] Volpe T, Kidner C, Hall I, Teng G, Grewal S (2002). Regulation of heterochromatic silencing and histone H3 lysine-9 methylation by RNAi. Science.

[pbio-0020133-Wassenegger1] Wassenegger M, Heimes S, Riedel L, Sänger H (1994). RNA-directed de novo methylation of genomic sequences in plants. Cell.

[pbio-0020133-Waterhouse1] Waterhouse P, Graham M, Wang MB (1998). Virus resistance and gene silencing in plants can be induced by simultaneous expression of sense and antisense RNA. Proc Natl Acad Sci U S A.

[pbio-0020133-Xie1] Xie Z, Johansen L, Gustafson A, Kasschau K, Lellis A (2004). Genetic and functional diversification of small RNA pathways in plants. PloS Biol.

[pbio-0020133-Zamore1] Zamore P, Tuschl T, Sharp P, Bartel D (2000). RNAi: Double-stranded RNA directs the ATP-dependent cleavage of mRNA at 21 to 23 nucleotide intervals. Cell.

[pbio-0020133-Zilberman1] Zilberman D, Cao X, Jacobsen S (2003). *ARGONAUTE4* control of locus-specific siRNA accumulation and DNA and histone methylation. Science.

